# Comparison of *Festuca glauca* ‘Uchte’ and *Festuca amethystina* ‘Walberla’ Varieties in a Simulated Extensive Roof Garden Environment

**DOI:** 10.3390/plants13162216

**Published:** 2024-08-09

**Authors:** Dóra Hamar-Farkas, Szilvia Kisvarga, Máté Ördögh, László Orlóci, Péter Honfi, Ildikó Kohut

**Affiliations:** 1 Department of Floriculture and Dendrology, Institute of Landscape Architecture, Urban Planning and Garden Art, Hungarian University of Agriculture and Life Sciences, 1114 Budapest, Hungary; farkas.dora@uni-mate.hu (D.H.-F.); kohut.ildiko@uni-mate.hu (I.K.); 2 Ornamental Plant and Green System Management, Institute of Landscape Architecture, Urban Planning and Garden Art, Hungarian University of Agriculture and Life Sciences, 1223 Budapest, Hungary; orloci.laszlo@uni-mate.hu

**Keywords:** *Festuca*, green roof, grasses, stress tolerance, urban green areas, ornamental plants

## Abstract

One of the most effective means of increasing urban green areas is the establishment of roof gardens. They have many positive properties and ecological functions, such as filling empty spaces with plants, protecting buildings, dust retention and air cleaning. In the case of extensive constructions, mostly *Sedum* species are used, planted as carpet-like “grass” sods or by installing modular units as plugs; however, with the use of other plant genera, the efficiency of ecological services could be increased by expanding the diversity. *Festuca* taxa have good drought resistance, and these plants tolerate temperature alterations well. Their application would increase the biodiversity, quality and decorative value of roof gardens. Experiments were carried out on nursery benches imitating a roof garden, with the use of modular elements intended for *Sedum* species, which facilitate the establishment of green roofs. In our trial, varieties of two European native species, *Festuca glauca* Vill. ‘Uchte’ and *F*. *amethystina* L. ‘Walberla’, were investigated. In order to find and determine the differences between the cultivars and the effects of the media (leaf mold and rhyolite tuff), we drew inferences after morphological (height, circumference, root weight, fresh and dry weight) and physiological tests (peroxidase and proline enzyme activity). We concluded that *F. glauca* ‘Uchte’ is recommended for roof garden conditions, planted in modular elements. Although the specimens were smaller in the medium containing fewer organic components than in the version with larger amounts, they were less exposed to the effects of drought stress. This can be a key factor for survival in extreme roof gardens or even urban conditions for all plants.

## 1. Introduction

### 1.1. The Importance of Ornamental Grasses in Settlements

In recent years, due to environmental problems and global changes, plants have received special attention due to their aesthetic properties and ability to improve the quality of their environment. A high degree of urban tolerance and increasing the area of green spaces with resistant plant species and ornamental varieties have also become important aspects [1]. For this reason, green areas have become a defining part of the urban ecosystem and environment [2]. Suitable grass species in cities have a large proportion of use as lawns and ornamental grasses [3,4]. The low maintenance and labor costs of ornamental grasses, as well as their high drought tolerance in many cases, become important [3]. In addition, the use of ornamental grass species increases the aesthetic value of the landscape and can also have a positive effect on biodiversity [5].

#### 1.1.1. The *Poaceae* Family and the *Festuca* Genus

Many members of the *Poaceae* family may be suitable for use in urban green areas, and, within this group, certain genera and species are highly resistant to urban stress factors. These grasses are also used in private gardens, green roofs and green walls [6] and they prefer soils with more nutrients [7]. However, the application of grasses on green roofs has not yet been studied thoroughly. The use value of ornamental grasses in green walls and roofs is also high from an aesthetic point of view, due to their unique size, color and plant form [2]. Due to their low maintenance requirements, they do not become frequent obstacles, even on roof parts that are relatively difficult to access. Additionally, these plants are also highly resistant to severe heat and drought stress [8].

In built-up, inhabited areas, *Festuca* taxa have been widely planted, and, regarding their use as ornamental grasses on green surfaces, the most important features are the foliage color, habitus and drought tolerance [9]. These low-growing, compact, mound-forming plants can have green, blue or golden leaves, and most are evergreen. In summer, they have delicate wand-like flowers. They grow in full sun, in free-draining soil that is moderately fertile to poor. These grasses are usually short-lived in damp, heavy or very fertile soil. The leaf color is better in sun than in shade [10]. Studies also recommend *Festuca* species for urban regions due to their optimal height, decorative appearance and hardiness [5]. *Festuca* have great impacts when planted en masse, providing a contemporary feel. The optimal environment for them consists of containers as well as borders [10]. Several species of the genus *Festuca* are important in urban applications [11]. *Festuca* occurs in the permeable soil of dry meadows, pastures, sands and rocky terrains in France, Belgium, Romania and Ukraine [12].

Another species, *F. arundinacea*, is also common in cities because of its good drought tolerance, so it can be an important, beneficial plant in urban use, in addition to grazing areas [13], as with *F. ovina* [14]. More ornamental varieties of *Festuca* species are becoming available, originating from biotechnological breeding [15], which can be a solution to increase drought tolerance [16].

#### 1.1.2. *Festuca amethystina* ‘Walberla’

*F. amethystina* is a native species in Central and South-Eastern Europe [11]. A mountain plant, it now occurs only as a relict [17].

Tufted *Festuca* is an evergreen, ornamental, cool-season grass with blue–green to blue–silver foliage and rolled and thread-like leaves and forms dense, attractive and evergreen tufts. When grown wild, it will reach 20–25 cm high with a similar width [18].

The flower stems grow above the foliage in summer, ending in small, green flower spikes with a pink–purple tint, becoming buff as the seeds ripen [19]. More ploidy-level types were described in Romania in 2019 [20]. It thrives in full sun and well-drained soil. Initially, regular and consistent watering is best, but, once established, it should need little water. It will tolerate light shade, but the foliage color will suffer with shade. It will also tolerate some drought and poor soil. It will not, however, grow well in poorly drained soil [18].

The variety of ‘Walberla’ is a dense, 20–40-cm-sized, bushy ornamental grass that produces soft leaves with red ends. It also prefers more sunshine or half-shaded areas and has excellent drought tolerance [21].

#### 1.1.3. *Festuca glauca* ‘Uchte’

*F. galuca* Vill. is a species used in floriculture, of which many types of ornamental grasses are known. It is very decorative due to its striking, silvery foliage color and attractive flowers [22]. These low-growing, compact, mound-forming plants can have green, blue or golden leaves, and most are evergreen. In summer, blue fescue produces spiky inflorescences [12]; from late spring to summer, it bears narrow, bristly blue–green leaves that turn golden brown [23].

In addition to its high decorativeness, it covers the ground throughout the year [24]. Its foliage color is inherited through the maternal line, which can play a major role in its breeding [25]. The species can also be used in roof gardens [26]. It is recommended to plant it in areas with northern exposure due to the lower mortality rate [24]. It grows best in full sun, but it also tolerates shade and semi-shade. It prefers well-drained soils; it has difficulty tolerating stagnant water, in which case it tends to rot in the middle [27].

*F. glauca* ‘Uchte’ is a compact, 20–30-cm-tall, blue–green grass with narrow, stiff, fibrous leaves. This extremely drought-tolerant plant prefers sunny, semi-shaded places and tolerates poor-quality soils [28].

#### 1.1.4. A Comparison of the Two Species Based on the Ellenberg Index Values

Based on the values in the Table 1, it is clear that the selected *Festuca* varieties can be suitable for use on green roofs.

### 1.2. Plant Application on Green Roofs

Green roofs are effective and natural ways to solve eco-environmental problems arising from climate change and rapid urbanization, as they provide multiple ecological services and have a significant impact on human well-being [32,33]. They have a significant effect in terms of cooling buildings and reducing energy consumption [34,35] and can also promote biodiversity in populated areas. The number of species that can be used on green roofs is limited [36]. In addition to the harsh climatic conditions, potential species must be hardened to the artificially created environment. Generally, the medium is not thick due to the loadability of the floor slab. Furthermore, strong solar radiation, extreme (usually too low but sometimes too high) precipitation and nutrient leaching also create difficulties for plants [37]. However, proper shading and media can increase the number of suitable species and varieties [36].

Nowadays, non-irrigated roof gardens play a major role, in which drought-tolerant plants [38], including ornamental and lawn species, become indispensable [39]. Grasses can be fully used as a continuous greensward on roof surfaces [40].

However, there are no standard assessment methods and tools for the evaluation of green roof ecosystem services; currently, there is still a lack of balance and coordination between different service values. Future research should focus on customizable, low-cost and innovative green roof designs, increasing the number of quantitative case studies and multi-perspective evaluations [32,33].

Although the plants for green roofs must be selected on the basis of their typology and morphology and the climatic conditions of the garden [41], vegetation with a very low water requirement is often considered [42]. In the case of an extensive roof garden, the vegetation layer mainly consists of drought-tolerant grasses and low-growing rock garden plants. They usually do not require regular care, except during the planting period [43]. Often, only *Sedum* species are used worldwide [41]. Compared to other species, *Sedum* is a very hardy genus; even with a thin substrate thickness, it tolerates the extreme climates of roof gardens and the poor nutrient content very well [44]. In drier climates, they are more advantageous for extensive planting, but it should not be overlooked that the performance of green roofs varies depending on the type of vegetation [45]. Most of its services and benefits are closely related to the environment and the vegetation layer [41].

### 1.3. The Responses of Ornamental Grass Species to Abiotic Stress

#### 1.3.1. The General Environmental Characteristics of Green Roofs

In urban environments, roof gardens are planted in very ecologically sensitive areas. Here, the plants have to survive in harsh conditions [37], which are not typical in their optimal living conditions, so they are not used to such extremes [46]. Due to the urban anthropogenic impact, the biological diversity has drastically decreased; the remaining habitats have become completely fragmented and degraded [47]; and the level of carbon dioxide, dust and heavy metal pollution is well above the average values [48]. In the absence of plants, the rain washes out the accumulated pollutants from the soil solution, which flow into the groundwater, deteriorating its quality [49]. Due to the large concrete surfaces, the temperature is extremely high, and due to heat radiation from the heating surfaces, the environment cannot cool down, even at night. Strong winds also often hinder the normal development of plants on roof gardens [50]. In addition to the harsh climatic conditions, many species must also be acclimated to the artificially created environment. The medium is often not thick enough, due to the load capacity of the slab, high solar radiation and low water supply, or it is sometimes covered with water for a long period of time, and the associated leaching of nutrients also poses difficulties for plants [37].

#### 1.3.2. The Stress Management of Grasses

Drought and heat are the two main sources of stress that are predicted to increase in the future due to climate change [51]. In nature, plants are simultaneously exposed to various biotic and abiotic stresses, and these stress effects adversely affect plant growth through changes in physiological and biochemical processes [52]. Roof gardens are more exposed to these negative effects [33]. Grasses are cosmopolitan in distribution, so their physiological responses to drought can differ greatly between species and populations [53]. In ornamental grasses, including the *Festuca arundinacea* species, the fresh and dried weight and chlorophyll content decrease during drought stress. The amount of proline and oxidase enzymes accumulated in the plant mitigate the harmful effects of drought, but *F. arundinacea* is less resistant to drought [54].

#### 1.3.3. The Role of POD and Proline Levels in Plant Stress Management

The drought tolerance of species classified within the same taxonomic category is similar [53]. Changes in gas and water exchange [55], as well as chlorophyll damage and impaired functioning of the photosynthetic electron transport chain [56], are important physiological processes in the response to stress. Through damage to photosynthetic processes, the thylakoid membrane and pigment formation become limited, which eventually causes cell death [57]. As a result of drought stress, the root diameter decreases, and the resulting vacuoles hinder the transport of water and nutrients [58]. The epithelial cells of festucoid grasses are capable of developing root hairs; thus, the cells have the same level of peroxidase activity in response to stress [59]. Water stress can cause significant changes in some metabolic factors, such as a decrease in chlorophyll content and the accumulation of proline. The high concentrations of free proline accumulation and sugar represent adaptive mechanisms in the plant under abiotic stress [60]. Several species can easily regenerate after drought stress. After drought, the physiology of plants regenerates to a large extent under the influence of irrigation. Proline and salicylic acid are actively involved in the fight against drought stress [53]. In the case of drought-stressed *Hordeum vulgare* plants (*Poaceae*), the shoot length, plant dry weight and chlorophyll content increased proportionally with the intensity of proline and salicylic acid. Proline accumulation was higher in stressed plants, due to the key role of proline in osmotic regulation under drought stress [57]. The accumulation of this amino acid (in addition to guaiacol and ascorbate) provides protection against oxidative damage and lipid peroxidation in *Festuca* species [61].

### 1.4. The Effect of the Planting Medium

The use of zeolites can improve the development properties of green roof plants and reduce the harmful effects of drought stress on grasses [62]. Farizani et al. [63] point out that the use of compost as a growing medium can improve the adaptation of less suitable grass species in the urban environment due to its high nutrient content and porosity. This may also facilitate the use of less drought-tolerant species. Composted green waste is also an important substrate because it can be produced locally in settlements [64]. Each type of green roof may require components with a different composition and ratio, because the decomposition of organic matter depends on several factors, such as the needs of the plant species, the climate or the exposure to the weather [65]. The combined use of compost and volcanic rocks can also be useful [66].

In the case of progressive soil desiccation, minimizing water loss and maximizing water uptake, biomass allocation is associated with strong root development in drought-avoidant species [67]. The root dry weight shows a significantly positive correlation with the plant height and dry weight of the aboveground green parts under drought stress [58]. The strength of leaf–stem vulnerability segmentation indicates that the leaves protect perennial rhizomes from severe drought stress [68].

Plants used on green roofs are exposed to extreme loads. Only a few genera tolerate these conditions. Further research can help to make green roofs even more diverse when they are installed, since grasses, including *Festuca* species, have wide tolerance. This is needed in the face of climate change and the increasing impacts of urbanization. In our research, we compare two commercial varieties in order to determine which is better able to withstand the extreme conditions of the roof garden.

## 2. Results

### 2.1. Morphological Studies

#### 2.1.1. Plant Height

**Figure 1:**
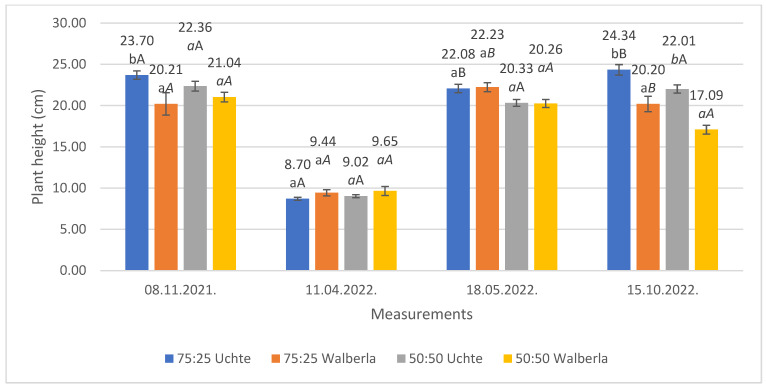
The average heights of the *Festuca* plants with the measurements. The letters indicate the significance levels, separated by measurement. ANOVA: 08 November 2021. By medium: 75:25: 0.021; 50:50: 0.121; by plant: ‘Uchte’: 0.096; ‘Walberla’: 0.583; 11 April 2022. By medium: 75:25: 0.088; 50:50: 0.274; by plant: ‘Uchte’: 0.213; ‘Walberla’: 0.750; 18 May 2022. By medium: 75:25: 0.840; 50:50: 0.911; by plant: ‘Uchte’: 0.011; ‘Walberla’: 0.010; 15 October 2022. By medium: 75:25: 0.001; 50:50: 0.000; by plant: ‘Uchte’: 0.006; ‘Walberla’: 0.006.

The height of the grasses (Figure 1) was almost completely equal, both at the time of planting and at the first measurement.

The drastic decrease in height between the two measurements was caused by the drying of the leaves (the dimensions were measured from the height of the new shoots). It was noted that the spring sprouting of the varieties on the same and different media was uniform. There were differences during the third and fourth measurements. In the case of the third occasion, the *F. glauca* ‘Uchte’ variety became significantly taller (22.08 cm) in the 75:25 medium than the individuals planted in the 50:50 medium (20.33 cm). A similar result was observed in the stock of *F. amethystina* ‘Walberla’, as the specimens grown in the 75:25 medium were considerably taller (22.23 cm) than the others in the 50:50 substrate (20.26 cm). At this time, we did not observe any differences between the varieties; only later (in the fourth examination) were differences between the media observed. The highest value (24.34 cm) was achieved by *F. glauca* ‘Uchte’ in the mixing ratio of 75:25, and *F. amethystina* ‘Walberla’ had the lowest value (17.09 cm) in the 50:50 mixture.

#### 2.1.2. Plant Circumference

**Figure 2:**
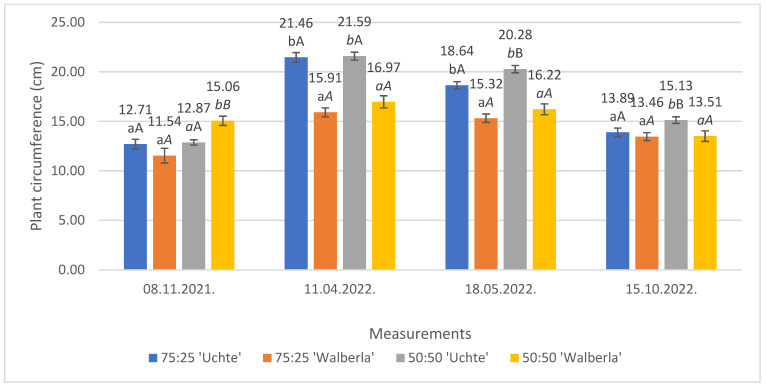
The average circumferences of the examined plants. The letters indicate the significance levels, separated by measurement. ANOVA: 08 November 2021. By medium: 75:25: 0.192; 50:50: 0.000; by plant: ‘Uchte’: 0.770; ‘Walberla’: 0.000; 11 April 2022. By medium: 75:25: 0.000; 50:50: 0.000; by plant: ‘Uchte’: 0.846; ‘Walberla’: 0.172; 18 May 2022. By medium: 75:25: 0.000; 50:50: 0.000; by plant: ‘Uchte’: 0.003; ‘Walberla’: 0.199; 15 October 2022. By medium: 75:25: 0.469; 50:50: 0.014; by plant: ‘Uchte’: 0.028; ‘Walberla’: 0.938.

Based on the data regarding the perimeter (Figure 2), it can be concluded that the perimeter of *F. glauca* ‘Uchte’ was greater than that produced the *F. amethystina* ‘Walberla’ variety.

The plants’ circle size decreased during the measurements, because, at the second examination, new, thick shoots increased the size of the base of the grass. Later, these organs were elongated; they thinned during subsequent growth; and the older leaves dried. We observed that this growth was more intense in the case of the *F. glauca* ‘Uchte’ variety, with thicker leaves than *F. amethystina* ‘Walberla’ (which developed thinner ones). At the time of the last measurement, except for the average of a variety planted in a medium with a ratio of 50:50, the sizes of the knots increased in the other media; thus, the grass population started to spread.

During the first measurement, the plant stocks grew evenly after planting, except for the *F. amethystina* ‘Walberla’ in the 50:50 mixture, where the development of the plants had started previously (15.06 cm). At the second investigation, after winter, new shoots emerged in the grass tufts, which increased the size of the base. There was no difference in growth between the media, and the medium containing less organic components (leafy mold) did not negatively affect the plants’ development. Comparing the varieties, a growth difference between *F. glauca* ‘Uchte’ (21.46 cm and 21.59 cm) and *F. amethystina* ‘Walberla’ (15.91 cm and 16.97 cm) was clearly visible. In the third examination, the reduction of the girth size was already observed, and, compared to the previous measurement, differences were detected between the *F. glauca* ‘Uchte’ varieties. In the 50:50 medium, *F. glauca* ‘Uchte’ had a significantly larger size (20.28 cm) than the specimens grown in the 75:25 mixture (18.64 cm). At the fourth monitoring, there was still a decrease in the average values of the plant stock, which mainly affected the ‘Uchte’ variety. The difference in the perimeter of the individuals of the *F. glauca* ‘Uchte’ growing in the 50:50 combination could be statistically verified compared to those that were grown in the other media.

#### 2.1.3. Root Length

The root length (Table 2) differences between the media were only obtained in the case of the *F. glauca* ‘Uchte’ cultivar.

These plants developed much shorter roots in the 75:25 medium (23.33 cm) than in the 50:50 mixture (32.87 cm). Examining the relationship between the varieties, we found a difference only in the latter substrate, in which case the variety *F. glauca* ‘Uchte’ exhibited outstanding values.

Regarding the circumference of the root (Table 2), we only found a difference between the varieties. In all cases, *F. glauca* ‘Uchte’ had a wider, larger root system than *F. amethystina* ‘Walberla’. The grittier medium texture did not affect the root circumference.

#### 2.1.4. Fresh and Dry Plant Weight and Moisture Content

Examining the fresh weight (Table 3), it was clear that *F. glauca* ‘Uchte’ grown on the 50:50 soil had the highest (161.47 g) and *F. amethystina* ‘Walberla’ had the lowest weight (66.74 g) in the case of the 75:25 mixture.

This was directly proportional to the values of the dry weight (Table 3); the plants lost almost the same amount of water during drying. In general, we observed that the same varieties achieved a higher total weight on average in the 50:50 soil than in the 75:25 mixture.

**Figure 3:**
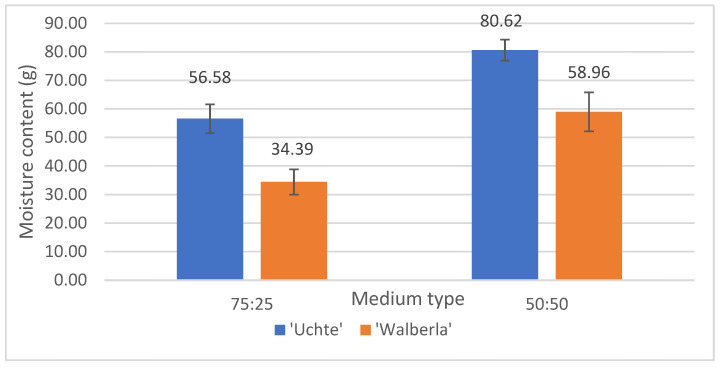
The moisture content of the plants (based on calculation with dry and fresh plant weight), separated by soil type.

According to the moisture content (Figure 3), the *F. glauca* ‘Uchte’ variety had the highest value (80.62 g) on the 50:50 soil.

Individuals of the *F. amethystina* ‘Walberla’ variety planted in the 75:25 mixture contained the least amount of moisture (34.39 g). A moisture difference between the media was observed; the water content of the plants grown in the 50:50 substrate was higher than that of the specimens planted in the 75:25 mixture.

### 2.2. Physiological Measurements

#### 2.2.1. Peroxidase

**Figure 4:**
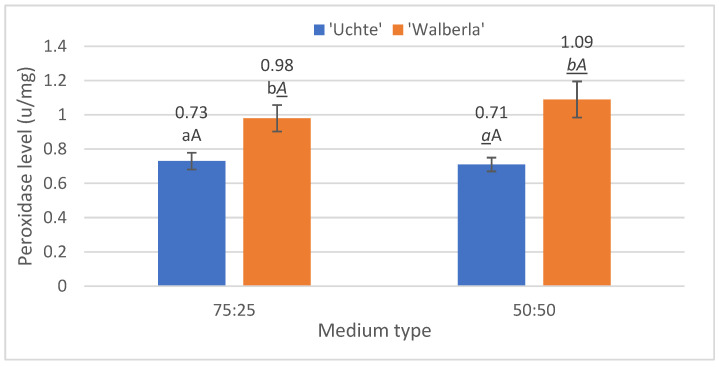
The peroxidase level per plant, separated by soil type. The letters indicate the significance levels. ANOVA. By medium: 75:25: 0.024; 50:50: 0.01; by plant: ‘Uchte’: 0.771; ‘Walberla’: 0.436.

During the peroxidase measurement (Figure 4), a difference between the soils was not detectable in the case of the plant varieties.

On the other hand, when examining the differences between the varieties, it was possible to demonstrate different tolerances for both types of media. On the 75:25 mixture, *F. glauca* ‘Uchte’ averaged 0.73 u/mg, while *F. amethystina* ‘Walberla’ averaged 0.98 u/mg. In the case of the 50:50 combination, *F. glauca* ‘Uchte’ did not show a significantly lower value, at only 0.71 u/mg, and *F. amethystina* ‘Walberla’ reached 1.08 u/mg.

#### 2.2.2. Proline

**Figure 5:**
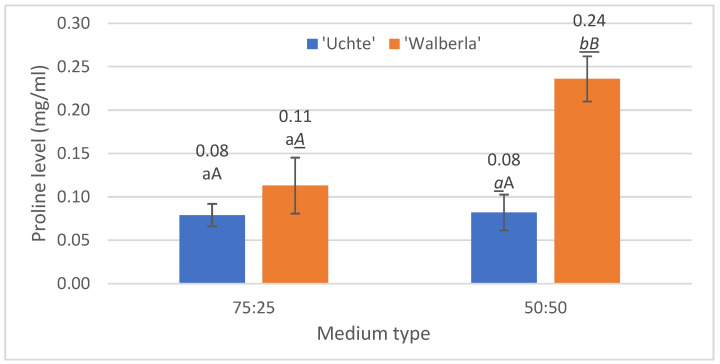
The proline level per plant, separated by soil type. The letters indicate the significance levels. ANOVA. By medium: 75:25: 0.375; 50:50: 0.01; by plant: ‘Uchte’: 0.898; ‘Walberla’: 0.041.

Investigating the proline content (Figure 5), a difference between the media was only detected in the *F. amethystina* ‘Walberla’ cultivar.

In this case, the plants were considerably stressed, twice as much, by the presence of the extra organic ingredient (leaf mold), while this stress effect was less typical in the medium mixture containing more mineral content (rhyolite tuff). The medium with higher organic matter content was more exposed to drying effects; it was able to retain less moisture during the drought period.

Taking into account the peroxidase and proline values, we also observed differences between the *Festuca* varieties, in the case of the use of the 50:50 mixture. *F. amethystina* ‘Walberla’ was affected by the conditions to a 1.5 times greater extent than the *F. glauca* ‘Uchte’ variety and was more stressed.

## 3. Discussion

Roof gardens are important and defining elements of today’s urban green space management, because, as Liu et al. [32] and Manso et al. [33] have stated, green roofs are an effective option to solve urbanization problems. As also described in the works of Van Der Kolk et al. [36], they can promote an increase in biodiversity in inhabited areas, even though the number of plant species that can be used there is relatively low. Due to the climatic extremes of recent years (severe drought periods, torrential rains and heatwaves), it has become important to find resistant plant species and varieties that tolerate such conditions [1]. The hardiness of the genus *Festuca* has been known for a long time. They tolerate drought and withstand stress well, and these grasses increase the biodiversity excellently, despite the fact that their maintenance costs are low [3,6]. As Gladkov et al. [2,8], Liu et al. [32] and Manso et al. [33] have also reported, the application of plants on green roofs is not yet a sufficiently researched subject area, so studies of this type can fill a gap regarding the green roofs of the future. Several species of the genus *Festuca* can potentially tolerate urban climates [5,13,14]. As Fodorpataki et al. [7] have described, grasses prefer soils with more nutrients, but Aguiar et al. [37] also note that nutrient leaching is common on green roofs. Therefore, it is worth investigating how the *Festuca* taxa behave in a nutrient-rich or poor environment. According to Kiedrzyński et al. [17], *Festuca amethystina* is a mountain species; it can tolerate lower nutrient content and can be promising for urban use. *Festuca glauca* is also suitable for green roofs, based on the recommendations of Yoon et al. [26], in addition to its extremely high decorative value; it beauty is also described by Oprea et al. [22] and Cojocariu et al. [24].

Compared with the height data, it could be clearly seen that the base of the grass tufts was wider in both varieties due to the new shoots, although it decreased with the increase in height; this was also described by Dossa [52]. It was observed that by the time of the third measurement, the individuals of the *F. amethystina* ‘Walberla’ variety had dried up to a large extent, while the individuals of the *F. glauca* ‘Uchte’ had better preserved their moisture. As a result, the circumferences of the plants increased by an average of 1 cm, except for *F. amethystina* ‘Walberla’ in the 50:50 medium, where the size and height of the plants decreased compared to the initial values.

Examining their roots, the variety *F. glauca* ‘Uchte’ grown in the 50:50 mixture substrate had the longest roots of the group, due to the higher rate of soil drying, as observed by Jiang and Huang [67]. In terms of circumference, however, the plants of this variety developed almost equally in the two types of media. Taking both types of data into account, the *F. glauca* ‘Uchte’ variety planted in the 50:50 medium produced the best root system. The root development of *F. amethystina* ‘Walberla’ was weaker in the roof garden conditions, regardless of the composition of the medium, which was probably induced by the stress caused by the prolonged drought. This can also be observed in the work of Chen et al. [58].

It can be concluded that *F. amethystina* ‘Walberla’ tolerated the roof garden conditions significantly less well than *F. glauca* ‘Uchte’. Although the 50:50 medium contained 25% more organic matter than the 75:25 substrate, it did not induce a significantly higher stress effect in our *Festuca* varieties, as observed by Farizani et al. [63] in their research.

However, laboratory tests showed that (as Bachle et al. [53] and Abdelaal et al. [57] also observed on other plants), in the 50:50 medium, the *F. amethystina* ‘Walberla’ specimens tolerated the conditions only with increased proline levels compared to *F. glauca* ‘Uchte’. Due to the composition of the medium, the water content of the plants planted in the 50:50 mixture was, on average, higher than that of the plants grown in the 75:25 soil. This did not help the plants, since the medium with higher organic matter content was more exposed to drying effects; it was able to retain less moisture during the drought period.

Based on the proline levels, therefore, in the drier period, the specimens of the 50:50 mixture tolerated the environment less well, with a greater stress effect, similarly to the study of Rai et al. [54]. High proline levels indicate high drought stress [53]. In the case of ‘Walberla’, the physiological processes were probably damaged to the extent described by Mur et al., Gao et al. and Chen et al. [55,56,58], who found that it was no longer able to regenerate after precipitation, as also mentioned by Bachle et al. [53].

The POD activity level of the *F. amethystina* ‘Walberla’ individuals was also much higher than in the other variety; however, this did not help the plant to tolerate the conditions better, as Abdelaal et al. [57] also found. There were no differences between the media within the species here, as stated by Krishnamurthy et al. [59].

## 4. Materials and Methods

During the experiment, 2 commercial varieties of the genus *Festuca*, namely *F. amethystina* ‘Walberla’ and *F. glauca* ‘Uchte’, were examined.

*F. amethystina* ‘Walberla’ is a dense, 20–40-cm-sized, bushy ornamental grass that produces soft leaves with red ends. It also prefers more sunshine or half-shaded areas and is drought-tolerant [21].

*F. glauca* ‘Uchte’ is a compact, 20–30-cm-tall, blue–green grass with narrow, stiff, fibrous leaves. This extremely drought-tolerant plant prefers sunny, semi-shaded places and tolerates poor-quality soils [28].

The plants were maintained under non-irrigated, extensive field conditions throughout the study period. The container plants were placed in the elements of the roof garden model on 27th October 2021. The simulated roof garden was located in an oblong rectangle, with an NE–SW orientation, which was not affected by any shading effects. A total of 42 plants were examined in the given cultivation year. Four measurements were taken during the experiment, and all experiments were repeated twice:

- 1st: condition assessment after planting, 10 November 2021;
- 2nd: sprouting after winter dormancy, 10 April 2022;
- 3rd: state before summer dormancy, 15 May 2022;
- 4th: culture finishing, elimination of the stock, 15 October 2022.

**Figure 6:**
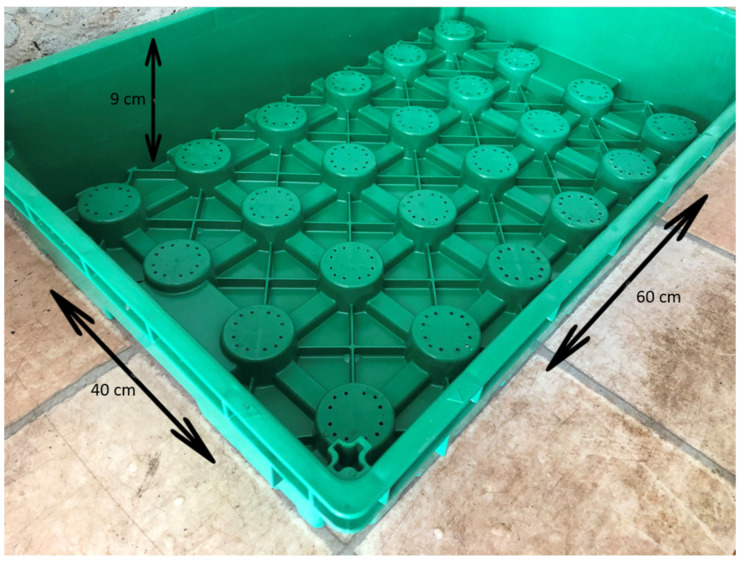
Modular green roof element developed by Fito System Kft.

The experiment was carried out on a 5 m × 1 m plant table that simulated a roof garden, at the Budatétény site of the Hungarian University of Agriculture and Life Sciences. The layering typical of roof gardens was ensured by agro-fabric and unique modular green roof elements developed by Fito System Kft [69]. The modular roof garden elements were plastic trays with an area of 40 cm × 60 cm and a side wall of 9 cm (Figure 6).

**Figure 7:**
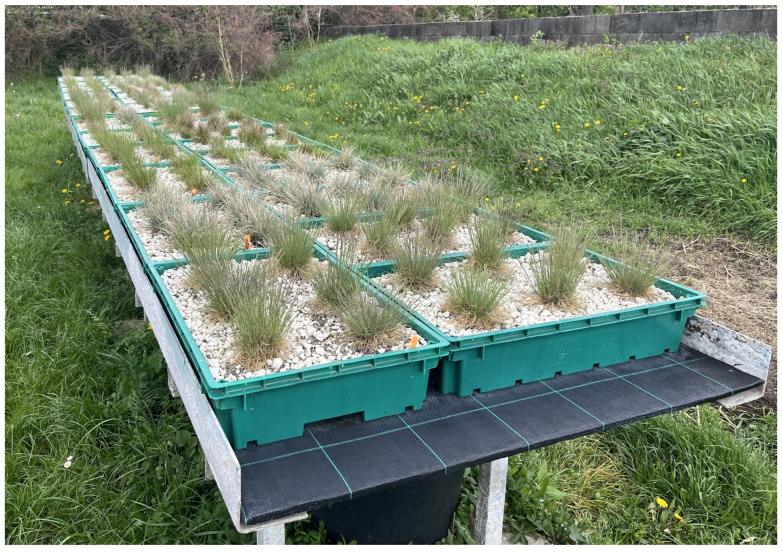
The table modeling the roof garden, which was located 100 cm above the ground.

During the imitation of the roof garden, we used two types of media components, mixed leaf mold and rhyolite tuff, which originated from Bodrogkeresztúr. The topsoil provided the organic material for the plants, and the rhyolite tuff with a particle size of 5–12 mm loosened it as an inorganic component, improved the soil structure and aided in water retention. The trays were divided into two parts; one half was filled with a 50:50 and the other half with a 75:25 soil mixture. In the latter, the amount of rhyolite tuff was increased. Then, the trays were again divided into 2 parts and the two types of plants were planted in equal proportions in both types of media mixture. Six plants were planted in each tray. The model table was located in an area exposed to full sunlight, with a horizontal design, at a height of 100 cm from the surface of the ground (Figure 7).

The samples for the laboratory tests were taken during the third measurement, before the summer dormancy began. Independent, random sampling was ensured during the investigation. During the measurements, the plant height and circumference were measured; during the fourth measurements, besides these, the root length and circumference, the fresh and dry plant weight and the moisture content were also analyzed. In the summer, laboratory tests were performed with the use of fresh leaves collected from stressed plants, in order to determine the chlorophyll and carotenoid content, peroxidase enzyme activity and proline level.

### 4.1. Measurement of Peroxidase Enzyme Activity

Peroxidase (POD) is a stress enzyme that is produced in plants as a result of stress [59]. With its accumulation, it can mitigate the harmful effects of drought [54], so it is important to examine its presence in plants. The activity of the POD enzyme was measured with a spectrophotometer and also with the use of frozen leaves, in the amount of 100 mg per group, with five repetitions, based on the methodology of Shannon et al. [70]. They were ground individually in an ice-cold mortar with a small amount of quartz sand plus 1200 μL of K-phosphate buffer solution at a temperature of 4 °C. The samples filled in the centrifuge tube were settled in the pre-cooled, 4 °C centrifuge for 20 min at a speed of 13,500 rpm. To measure the color reaction, a concentrated 30% H_2_O_2_ solution diluted 100× was used, and orthodianizidine (3,3′-dimethoxybenzidine) was dissolved in methanol with a concentration of 10 mg/mL. The buffer was 4 °C Na-acetate. The blank sample consisted of 30 μL H_2_O_2_, 20 μL orthodianizidine and 1700 μL Na-acetate. For plants’ POD activity analysis, 50 μL leaf samples were mixed with 30 μL H_2_O_2_, 20 μL orthodianizidine and 1650 μL buffer. After shaking, the photometer measured the change in absorbance every 10 s, from which the enzyme activity could be calculated (1):

enzyme activity = (ΔA1 × dilution)/ɛ [unit/mL, U/mL](1)

where ΔA1 = absorbance change in less than 1 min; ɛ = 11.3: extinction coefficient of orthodianizidine (characterizes the degree of color change). This can be converted to units/mg (2):

(unit/mL) × (w/V)(2)

where V = amount of tissue extract (1.5 mL); w = weight of fabric (~0.1 g).

### 4.2. Proline Measurement

Proline accumulates in large amounts in stressed plants. In the presence of drought stress, it plays a key role in osmotic regulation [57], which helps plants to survive physiologically stressful periods [54]. Proline was determined according to the methodology of Ábrahám et al. [71]. From the frozen samples, 100 mg of plant parts was weighed and rubbed with a solution containing 3% sulfosalicylic acid, using quartz sand (5 µL/mg fresh weight). The extracts were sedimented for 10 min at a speed of 14,000 rpm, and 100 µL of the supernatant was measured per sample, with three repetitions. For this, 200 µL of 96% acetic acid and 200 µL of acid ninhydrin (2.5% (*w*/*v*) ninhydrin, 60% (*v*/*v*) 96% acetic acid, 40% (*v*/*v*) 6M phosphoric acid) were added. The test tubes containing the mixtures were covered with aluminum foil and heated in an oven at 96 °C for one hour. The reaction was stopped after one hour in cold water containing ice. After this, the samples were extracted with 1.5 mL of toluene. The dissolution was facilitated by vortexing for approximately 20 s, after which all samples were left to rest for 5 min. The absorbance of the red-colored supernatants was determined in a narrow cuvette and examined at 520 nm. The values were compared to a calibration curve prepared using an α-proline concentration series containing a known amount of proline.

### 4.3. Data Evaluation

The cultivars *F. amethystina* ‘Walberla’ and *Festuca glauca* ‘Uchte’ were examined, and these plants were owned by the Hungarian University of Agriculture and Life Sciences. The processing, comparison and examination of the measurable deviations of our results were carried out with the IBM SPSS Statistics 26 program, using the ANOVA method. In all cases, the measured data were analyzed at a 95% reliability (significance) level. Having evaluated the Levene test, if the Sig. > 0.05, the Tukey test was applied, and if Sig. < 0.05, the Games–Howell post hoc test was applied. The Games–Howell test and the Tukey test were applied as a function of the data series. If the homogeneity of the data was violated, the Games–Howell test was applied, as this test is suitable for the ranking of data accordingly and calculating the correct statistical results. If the homogeneity was not violated, the Tukey test was applied.

## 5. Conclusions

Green roofs help to maintain biodiversity, both among plants and animals. The modular roof garden elements used in the experiment are also suitable for other plants and not only *Sedum* taxa. Further experiments would help us to offer new, alternative options when installing green roofs. There are many possibilities with a wide range of grasses, and the studied *Festuca* varieties tolerated the roof garden conditions well. The decorative *F. glauca* ‘Uchte’ proved to be more suitable for planting, as it was much less stressed than *F. amethystina* ‘Walberla’. Based on the two types of medium, it can be said that although the plants generally grew larger in the medium containing more organic matter (leaf soil, also known as leaf mold), they were more exposed to the effects of drought stress. However, in severe or extensive roof garden conditions, a larger height is not necessarily more advantageous for plants. In drier periods, it is associated with the more intense re-drying of the leaves, making them less attractive, which is an essential element of roof gardens. Thus, comparing the two varieties, the *F. glauca* ‘Uchte’ variety is recommended to be planted in a medium with less organic matter in extensive (non-irrigated) conditions. This variety is able to withstand harsh roof garden conditions, even in environments with less nutrients, and can serve as an outstanding and defining element of the green roof, with lower maintenance costs. Climate change and urbanization are an accelerating global problem to which we must respond. A good option for plant professionals is to use plants that can be used on urban green spaces with a small budget. These green areas offer an alternative to green roofs. Taking into account the ecological needs, botanical characteristics, ornamental and horticultural varieties and decorativeness indicators, it is important review the selection of varieties for these alternative surfaces as well. Since the base species of the studied varieties are widely used throughout most of the world, according to the results of the experiment, they can be used not only in the European urbanized environment but also on other continents, worldwide, in many cities. This is a promising result that could bring green space management researchers, ecologists and gardeners closer to creating a more sustainable world.

## Figures and Tables

**Table 1:** Comparison of applied *Festuca* species based on the Ellenberg index [29,30,31]. Light indicator value: 1–9 from deep shadow to bright light. Temperature indicator value: 1–9 from colder temperature tolerance to warmer climates. Moisture indicator value: 1–12 from plants that tolerate more drought to plants that require water coverage. Nutrient indicator value: 1–9 from the tolerance of soils poorer in nutrients to the need for soils rich in nutrients.

Indicator Value	Light	Temperature	Moisture	Nutrients
*F. amethystina*	6	5	3	2
*F. glauca*	8	9	5	5

**Table 2:** The average length and circumference of the roots, separated by soil type. The letters indicate the significance levels. ANOVA. Root length by medium: 75:25: 0.968; 50:50: 0.09; by plant: ‘Uchte’: 0.000; ‘Walberla’: 0.059. Root circumference by medium: 75:25: 0.000; 50:50: 0.000; by plant: ‘Uchte’: 0.367; ‘Walberla’: 0.660.

	75:25 ‘Uchte’	75:25 ‘Walberla’	50:50 ‘Uchte’	50:50 ‘Walberla’
Root length (cm)	23.33 ± 1.26 ^aA^	23.40 ± 1.27 ^a*A*^	32.87 ± 1.96 ^*a*^^B^	26.70 ± 1.13 ^*bA*^
Root circumference (cm)	31.44 ± 1.00 ^bA^	21.91 ± 0.95 ^a*A*^	30.32 ± 0.71 ^*b*^^A^	22.44 ± 0.70 ^*aA*^

**Table 3:** The fresh and dry weights of plants, separated by soil type. The letters indicate the significance levels. ANOVA. Fresh weight by medium: 75:25: 0.000; 50:50: 0.000; by plant: ‘Uchte’: 0.001; ‘Walberla’: 0.007. Dry weight by medium: 75:25: 0.000; 50:50: 0.000; by plant: ‘Uchte’: 0.002; ‘Walberla’: 0.011.

	75:25 ‘Uchte’	75:25 ‘Walberla’	50:50 ‘Uchte’	50:50 ‘Walberla’
Fresh plant weight (g)	115.36 ± 10.27 ^bA^	66.74 ± 7.54 ^a*A*^	161.47 ± 7.69 ^*b*^^B^	105.28 ± 11.16 ^*aB*^
Dry plant weight (g)	58.78 ± 5.23 ^bA^	32.35 ± 3.13 ^a*A*^	80.85 ± 4.00 ^*b*^^B^	46.32 ± 4.33 ^*aB*^

## Data Availability

Dataset available on request from the authors.
